# Rationale and Design of a Telehealth Self-Management, Shared Care Intervention for Post-treatment Survivors of Lung and Colorectal Cancer

**DOI:** 10.1007/s13187-021-01958-8

**Published:** 2021-01-08

**Authors:** Virginia Sun, Anne Reb, Marc Debay, Marwan Fakih, Betty Ferrell

**Affiliations:** 1 Department of Population Sciences, City of Hope, Duarte, CA USA; 2 Department of Surgery, City of Hope, Duarte, CA USA; 3 Department of Family Medicine, University of California, Riverside, CA USA; 4 Department of Medical Oncology and Therapeutics Research, City of Hope, Duarte, CA USA

**Keywords:** Cancer survivorship, Lung cancer, Colorectal cancer, Self-management, Shared care, Self-efficacy, Primary care

## Abstract

Survivors of lung and colorectal cancer have high post-treatment needs; the majority are older and suffer from greater comorbidities and poor quality of life (QOL). They remain underrepresented in research, leading to significant disparities in post-treatment outcomes. Personalized post-treatment follow-up care and care coordination among healthcare teams is a priority for survivors of lung and colorectal cancer. However, there are few evidence-based interventions that address survivors’ post-treatment needs beyond the use of a follow-up care plan. This paper describes the rationale and design of an evidence-informed telehealth intervention that integrates shared care coordination between oncology/primary care and self-management skills building to empower post-treatment survivors of lung and colorectal cancer. The intervention design was informed by (1) contemporary published evidence on cancer survivorship, (2) our previous research in lung and colorectal cancer survivorship, (3) the chronic care self-management model (CCM), and (4) shared post-treatment follow-up care between oncology and primary care. A two-arm, parallel randomized controlled trial will determine the efficacy of the telehealth intervention to improve cancer care delivery and survivor-specific outcomes. **ClinicalTrials.gov****Identifier:** NCT04428905

## Introduction

More than 16 million Americans, most over age 65, are living with a history of cancer [1]. Post-treatment unmet healthcare needs are common, particularly for survivors of lung and colorectal cancer. Our previous research and those from the published literature suggest that lung and colorectal cancer survivors suffer from persistent long-term effects of treatment, such as dyspnea, fatigue, pain/neuropathy, bowel dysfunction, ostomy care, anxiety, depression, and distress. These symptoms contribute to a dramatic deterioration in post-primary treatment quality of life (QOL) [2–8].

Lung cancer is the second most common cancer diagnosis in the USA, and more than 570,000 Americans are living with a history of lung cancer [9]. Median age at diagnosis is 70. Due to advances in early detection and treatment, lung cancer survivors are living longer [10]. Treatment-induced late and long-term effects are common and include radiation pneumonitis, pulmonary fibrosis, post-thoracotomy pain syndrome, chemotherapy-induced peripheral neuropathy, fatigue, dyspnea, cough, and immune-related adverse events [11, 12]. Up to 50% of survivors experience impaired pulmonary function [13]. Between 11 and 44% of survivors report psychological distress and depression post-treatment, and these are often severe enough to require intervention [14]. As a result of tobacco exposure, survivors of lung cancer have an increased risk for smoking-related comorbidities, including chronic obstructive pulmonary disease and cardiovascular disease [15]. Survivors who are current and former smokers are at risk for developing a second lung cancer and other smoking-related cancers, particularly in the head and neck and urinary tract [16].

An estimated 1.5 million Americans are living with a history of colorectal cancer, and three-quarters (76%) are 65 years and older [17]. Recent trends suggest that rates of colorectal cancer are increasing in adults 55 years and younger [18]. These trends suggest that the number of younger survivors of colorectal cancer will continue to increase in the years to come. Common late and long-term effects of treatment include oxaliplatin-induced peripheral neuropathy, bladder dysfunction, sexual dysfunction, and chronic diarrhea [19, 20]. Bowel dysfunction is common among survivors of rectal cancer and is associated with frequent and erratic bowel movements, fecal incontinence, soiling, gas, bloating, and oscillations between diarrhea and constipation [21, 22]. Persistent bowel symptoms lead to reduced social activities, poor emotional well-being, and decrements in QOL [23]. A permanent colostomy may be required in 29% of survivors of rectal cancer [24. Permanent colostomies represent a major life adjustment and often result in financial toxicity due to lifelong need to pay for ostomy supplies [25]. Negative body image leads to social isolation, psychological distress, and depression [20]. Survivors’ adherence to healthy lifestyle behaviors (diet, physical activity) is inadequate and leads to increased risk of cancer recurrence and obesity-related comorbidities [24].

In addition to QOL concerns, post-treatment care challenges are compounded by the growing complexity of cancer care and fragmented follow-up. A potential solution to the post-treatment care challenge is through advanced practice nurse (APRN)-led follow-up care that promotes survivor self-management skills training. In addition, the APRN-led follow-up care includes care coordination between oncology and primary care to address surveillance, comorbidity management, and healthy living/preventive care. The purpose of this paper is to describe the rationale, development, and design of a telehealth self-management, shared care intervention for post-treatment survivors of stages I–III lung and colorectal cancer.

## Intervention Overview

### Conceptual Frameworks

Intervention design is guided by the chronic care self-management model (CCM) [26–28]. Self-management is defined as “the systematic provision of education and supportive interventions to increase survivors’ skills and confidence in managing their health problems, including goal setting and problem-solving” [29]. The CCM transforms a reactive health system into one that improves survivor outcomes through proactive planning and building self-efficacy. Central to self-management is the concept that chronic illness management is a partnership between survivor and the healthcare team. Together, the partners identify goals that are important to post-treatment follow-up care and develop realistic action plans to meet those goals [29]. Needs and goals are systematically and consistently addressed through productive interactions between survivors and the healthcare team [29]. A central construct of self-management is self-efficacy, defined as confidence to carry out behaviors necessary to achieve a desired goal [30]. Self-efficacy is enhanced when survivors succeed in building confidence in their ability to manage their follow-up care.

Several well-established behavioral approaches are included in the intervention. Survivors are coached to identify and set short- and long-term goals in relation to their post-treatment care. Specific, measurable, attainable, realistic, and time-bound (SMART) goal setting provides clarity for survivors and APRNs in terms of structure and expected outcomes [31]. Goal setting is combined with self-monitoring of late and long-term effects to promote self-efficacy in symptom management, comorbidity management, and maintenance of physical functioning [31]. The APRNs work with survivors to identify potential challenges and problems to self-management; it is key that survivors identify the challenges as well as potential solutions rather than the APRN. Ultimately, the survivor and the APRN define new realistic and adoptable strategies to achieve the best post-treatment follow-up care and QOL possible.

The intervention is also guided by Nekhlyudov and colleagues’ quality of cancer survivorship care framework [32]. There are five quality survivorship care domains within the framework, and these include surveillance/management of physical effects, psychosocial effects, comorbidities, prevention and surveillance for recurrence and new cancers, and health promotion/disease prevention. These domains are the key content for self-management skills building component of the intervention. The clinical care structure for the intervention is APRN-driven and disease-specific (lung, colorectal). This structure was selected because APRNs have adequate training and skills to address follow-up care issues [33]. In follow-up care, APRNs often serve as care coordinators and the communication bridge between multiple specialties. In addition, APRNs are heavily embedded within oncology care structures, practicing alongside oncologists in all settings. The shared care approach focuses on the collaboration between oncology and primary care and leverage the expertise of each specialty to address the survivor’s needs within the five quality domains. The technology platform leverages telehealth (videoconferencing) for intervention delivery, which can potentially decrease travel burden, increase scalability/sustainability, and reduce burden on institutional clinic space.

### Intervention Content

The intervention is an evidence-informed, APRN-driven, self-management shared care model of personalized cancer follow-up care. It includes the following components: (1) care coordination and communication between oncology and primary care on health promotion, cancer prevention, and comorbidity management; (2) provision of a clinician care plan for PCPs; (3) comprehensive patient/geriatric assessment; (4) personalized follow-up care plan for survivors; and (5) survivor self-management skills building for surveillance/follow-up, late and long-term effects of treatment, and comorbidities.

The intervention is delivered through five telehealth, videoconferencing sessions over a 4-month period, followed by three monthly maintenance telehealth sessions (see Table 1). Each session lasts approximately 30–60 min. Prior to initiating the telehealth sessions, the APRN collaborates with the oncology team and generates a personalized surveillance/follow-up plan for each participant. Using baseline patient-reported outcomes (PROs) on QOL and patient/geriatric assessment, the APRN develops a personalized follow-up care plan. The geriatric assessment is administered to survivors of all ages because many of the constructs (social support, functional status) are relevant regardless of age.

**Table 1:** Intervention content

Component	Content
Before sessions	• APRN and oncologist generate personalized surveillance/follow-up plan • APRN complete personalized survivorship care plan/resource manual using baseline PROs and patient/geriatric assessment
Session 1	• APRN finalize and review care plan/resource manual with survivors • SMART goal setting • PCP identification • APRN complete brief (2 page) clinician survivorship care plan and send to PCP • Review shared care responsibilities with PCP • Clinic encounter notes exchange between oncology and primary care
Sessions 2 and 3 Late/long-term effects and comorbidity management	• Review care plan/resource manual • Discuss physical, emotional, social, and spiritual well-being concerns • Discuss signs and symptoms of recurrence • Discuss comorbidity management • Identify challenges; discuss strategies to overcome challenges through problem-solving • Review institutional/community resources • Initiate referrals as needed • Review and assess SMART goals • PCP engagement on shared care responsibilities
Sessions 4 and 5 Physical functioning and healthy living	• Review care plan/resource manual • Discuss maintaining physical functioning • Discuss healthy living recommendations • Identify challenges; discuss strategies to overcome challenges through problem-solving • Provide institutional/community resources • Initiate referrals as needed • Review and assess SMART goals • PCP engagement on shared care
Monthly maintenance sessions	• Review self-management skills, SMART goals, and care plan/resource manual • PCP engagement on shared care • APRN recommendation on possible full transition of care to PCP, or continue with shared care • Discussion with survivors on preference for full transition or continued shared care
APRN (throughout the study)	• Communication and care coordination between oncologist and PCP

For survivors with an identified PCP, the APRN initiates communication and care coordination with the PCP team through email and telephone. A brief, two-page clinician follow-up care plan is emailed or mailed to the PCP. The brief clinician-focused care plan contains information on the PCP’s care responsibilities, which includes (1) health promotion (diet, physical activity, alcohol use, sun protection, etc.); (2) vaccinations/other cancer screening; and (3) comorbidity management. The APRN reviews the clinician care plan with the PCP and discusses shared care responsibilities by telephone. Secure exchange of oncology and PCP clinic visit notes is coordinated by the APRN and entered into the cancer center’s electronic health record (EHR). All survivors receive a comprehensive resource manual that contains session content and additional resources.

In telehealth session 1, the APRN reviews the personalized follow-up care plan with the survivor to tailor the follow-up care plan based on needs and preferences. SMART goals of follow-up care are identified. In sessions 2 and 3, late/long-term effects of treatment and comorbidity management are discussed. The APRN coaches survivors on managing physical (including signs and symptoms of recurrence), emotional, social, and spiritual well-being issues. Comorbidities are reviewed, and self-management strategies are discussed; this includes a focus on the care responsibility of different healthcare teams (oncologist versus PCP). Survivors identify unique and relevant challenges related to late/long-term effects and comorbidity management and develop an action plan to overcome the challenges. Together, the APRN and survivor identify potential institutional and community resources. SMART goals are reviewed and revised.

Sessions 4 and 5 focus on physical functioning and healthy living. Specifically, survivors are coached on strategies to maintain physical functioning. Healthy living recommendations (diet, physical activity, tobacco cessation) are introduced and discussed. This includes (1) identification of perceived barriers to behavior change, (2) prior plans or strategies to overcome these barriers, and (3) identification of new strategies that are adoptable to promote health behaviors. Functional capacity (falls prevention, gait, balance) is reviewed. Institutional and community resources are identified, and referrals initiated (i.e., rehabilitation services) as needed. SMART goals are reviewed and revised as needed.

Three monthly telehealth maintenance sessions are administered following the completion of session 5. In these sessions, the APRN reviews progress on the survivors’ self-management skills building and provides additional coaching as needed. SMART goals and personalized follow-up care plans are reviewed and revised as needed. Care coordination and communication with PCPs continue regularly during the maintenance session months.

## Discussion

A recent National Academy of Science Workshop found that current models of follow-up care fail in meeting the needs of survivors, despite decades of progress in research [34]. Limited evidence exists on the efficacy of follow-up care approaches. Focused efforts are needed to improve care delivery and increase adherence to long-term treatment and follow-up care guidelines. Hybrid approaches may facilitate communication and care coordination among primary care, oncology, and survivors [35].

The anticipated aging of the US population will increase the complexity of cancer care, because older adults are more likely to have higher non-cancer specific needs (functional declines, comorbidities) in addition to cancer-specific needs [36]. Nearly 64% of cancer survivors are 65 years and older, and this number is estimated to rise to 73% by 2040 [17]. The presence of comorbidities, decline in organ function or physiologic reserves, and increased need for assistance with daily function complicates the care of older survivors [37]. Multimorbidity is one of the greatest challenges in caring for older survivors; > 50% have three or more comorbidities (e.g., cardiovascular, pulmonary) [38]. Multimorbidity is associated with treatment-related adverse events, poorer outcomes, and challenges in care coordination due to complexity of care [15].

For older cancer survivors, comprehensive geriatric assessment can improve the quality of follow-up care. Geriatric assessment is used to understand the heterogeneity of the aging process through an evaluation of a survivor’s (1) functional status, (2) comorbidities, (3) cognition, (4) psychological status, (5) social functioning and support, (6) medication review, and (7) nutritional status [39]. Each of these domains is an independent predictor of morbidity and/or mortality in the geriatric population. The comprehensive assessment can improve outcomes for older survivors by identifying areas of vulnerability in need of targeted interventions [40].

In most cancer care settings, survivor follow-up care is largely provided by oncologists. Survivors prefer to be followed by oncologists due in part to the lack of follow-up care expertise in non-oncology providers (i.e., PCPs). Survivors also have a higher level of emotional trust with oncologists that was developed during treatment [41]. From the oncologist’s perspective, seeing patients that are doing well after treatment may reduce work-related stress and burnout and contribute to higher job satisfaction [36]. Full transition of all survivors to PCP care is also impossible and likely inappropriate, due to a lack of formal training on follow-up cancer care and lack of time [42]. The anticipated shortage of the oncology workforce has resulted in greater delegation of complex cancer care to oncology advanced practice nurses (APRNs), who are also experiencing workforce shortages [43]. Robust evidence suggests that improved health outcomes are associated with oncology APRN-led care [44–46]. Hence, oncology APRNs are in an optimal position to support cancer survivors’ self-management skills building, coordinate care with a PCPs, and use telehealth effectively for post-treatment follow-up.

The shared care model can free up busy oncology clinics, eliminate the need to transition all survivors to busy PCPs, provide efficient and timely care coordination with the oncologists when needed, and promote personalized survivor-centered follow-up care [36]. Shared care is likely most appropriate for lung and colorectal cancer survivors with multiple complex needs and low to moderate levels of risk for recurrence, secondary cancers, symptom burden, functional declines, and comorbidities. Evidence from other countries suggests that in breast cancer survivors, the personalized approach improves receipt of timely follow-up screening, decreased waiting time to receive oncology care, and freed up clinician time by shifting resources to survivors with more complex needs [47]. Implementation of the shared care model and a personalized follow-up approach will likely be more complex in the USA due to diverse care delivery systems. The APRN-led care coordination and communication can potentially reduce care fragmentation.

For cancer survivors, treatment completion does not indicate an end to their cancer experience [48]. Ongoing relationships with the healthcare system are needed to manage post-treatment care and require long-term planning rather than acute, prescriptive relationships [29]. Being adequately prepared for post-treatment self-management can empower cancer survivors and improve their confidence in managing their overall healthcare [49, 50]. For cancer survivors, self-management skills building should include coaching patients on signs and symptoms of recurrence, cancer prevention, post-treatment symptoms, and comorbidities [36]. The National Academy of Medicine (formerly the Institute of Medicine) defines self-management support as the “systematic provision of interventions to increase skills and self-efficacy in managing health problems, including regular assessment of problems, goal-setting, and problem-solving support” [51]. Current evidence suggests that cancer survivors with low self-efficacy report worse physical and psychological well-being [50]. This suggests that interventions targeted at increasing self-efficacy and self-management may be beneficial for this population. Two randomized trials of self-management interventions reported trends toward greater self-efficacy, improved general health, and greater provider implementation of recommended care in breast cancer survivors [52, 53]. Effective self-management interventions can transform a reactive healthcare system into one that improves survivor outcomes through proactive planning [54].

In addition to addressing survivor needs, self-management skills building is a key component of personalized cancer follow-up care [49]. In the UK and Australia, where survivorship care is a national initiative, preliminary evidence points to self-management skills training as the driving factor for personalized follow-up based on risk [55]. The evidence shows that up to 50% of colorectal cancer survivors who were treated with curative intent were able to self-manage after treatment completion [56]. Supporting survivors’ self-management skills may free up oncology clinic space and time and reduce burden on the healthcare system [36, 55]. There is a need to test full-scale post-treatment self-management interventions and evaluate similar outcomes in the US healthcare system.

## Conclusions

There are few conceptually based, evidence-informed interventions in post-treatment follow-up care for survivors of lung and colorectal cancer. We propose that models of post-treatment follow-up care should incorporate oncology/PCP shared care and self-management skills coaching to empower and enable cancer survivors. Research is needed to identify the best approaches to support self-management of follow-up care and ongoing assessment and monitoring for late and long-term effects. Our team is currently conducting a randomized controlled trial of an APRN-driven telehealth intervention on healthcare team knowledge of follow-up care, care coordination between oncologist/PCP, communication across medical disciplines, and survivor-specific outcomes (confidence in follow-up care, QOL).

## Data Availability

NA
