# Correction to: Rationale and Design of a Telehealth Self-Management, Shared Care Intervention for Post-treatment Survivors of Lung and Colorectal Cancer

**DOI:** 10.1007/s13187-021-01969-5

**Published:** 2021-02-23

**Authors:** Virginia Sun, Anne Reb, Marc Debay, Marwan Fakih, Betty Ferrell

**Affiliations:** 1 Department of Population Sciences, City of Hope, Duarte, CA USA; 2 Department of Surgery, City of Hope, Duarte, CA USA; 3 Department of Family Medicine, University of California, Riverside, CA USA; 4 Department of Medical Oncology and Therapeutics Research, City of Hope, Duarte, CA USA

**Correction to: Journal of Cancer Education**

10.1007/s13187-021-01958-8

The article Rationale and Design of a Telehealth Self-Management, Shared Care Intervention for Post-treatment Survivors of Lung and Colorectal Cancer, written by Virginia Sun, Anne Reb, Marc Debay, Marwan Fakih and Betty Ferrell was originally published electronically on the publisher’s internet portal on 08 January 2021 without open access. With the author(s)’ decision to opt for Open Choice the copyright of the article changed on February 2021 to © The Author(s) 2021 and the article is forthwith distributed under a Creative Commons Attribution 4.0 International License, which permits use, sharing, adaptation, distribution and reproduction in any medium or format, as long as you give appropriate credit to the original author(s) and the source, provide a link to the Creative Commons license, and indicate if changes were made. The images or other third party material in this article are included in the article’s Creative Commons licensee, unless indicated otherwise in a credit line to the material. If material is not included in the article’s Creative Commons license and your intended use is not permitted by statutory regulation or exceeds the permitted use, you will need to obtain permission directly from the copyright holder. To view a copy of this license, visit http://creativecommons.org/licenses/by/4.0.

The original article has been corrected.

